# Retrosacral Morel-Lavallée lesion: resolution with ultrasound-guided drainage and sclerotherapy

**DOI:** 10.1259/bjrcr.20190120

**Published:** 2020-05-06

**Authors:** Samuel Edward Thomas Leach, Mark Wotherspoon, Leonard King

**Affiliations:** 1 Department of Radiology, Salisbury NHS Foundation Trust, Salisbury, United Kingdom; 2 Hampshire Hospitals NHS Foundation Trust, Basingstoke, United Kingdom; 3 Department of Radiology, University Hospitals, Southampton, United Kingdom

## Abstract

Morel-Lavallée lesions are chronic seromas due to closed degloving injuries, resulting from blunt trauma. They most commonly occur over the greater trochanteric, gluteal and flank regions. We present a case of retrosacral Morel-Lavallée lesion. Initial ultrasound demonstrated a fluid collection lying between the subcutaneous fat and the underlying fascia superficial to the sacrum. Following repeated ultrasound-guided aspirations, further recurrence of a superficial pre-sacral seroma was confirmed with MRI. Ultrasound-guided aspiration was performed and 100 mg of injectable doxycycline was instilled into the lesion. 4 months after sclerotherapy, the patient was asymptomatic, and follow-up MRI demonstrated no residual fluid collection or complication. This case demonstrates the value of using MRI in conjunction with ultrasound to characterize Morel-Lavallée lesions in an atypical site and in confirming response to treatment, in addition to the use of sclerotherapy for treatment of a lesion refractory to repeated aspiration.

## Clinical presentation

We present a case of retrosacral Morel-Lavallée lesion in a 36-year-old female who presented to a Sports Medicine clinic 3 weeks after incurring direct trauma to the lower back and pelvis due to a fall from a horse. She complained of persisting pain and soft tissue swelling in the lower lumbar and gluteal region extending into the natal cleft. Clinical examination revealed an ill-defined, tender, fluctuant swelling over the lower lumbar region and she was referred to radiology for further investigation.

## Imaging findings

**Figure 1.**
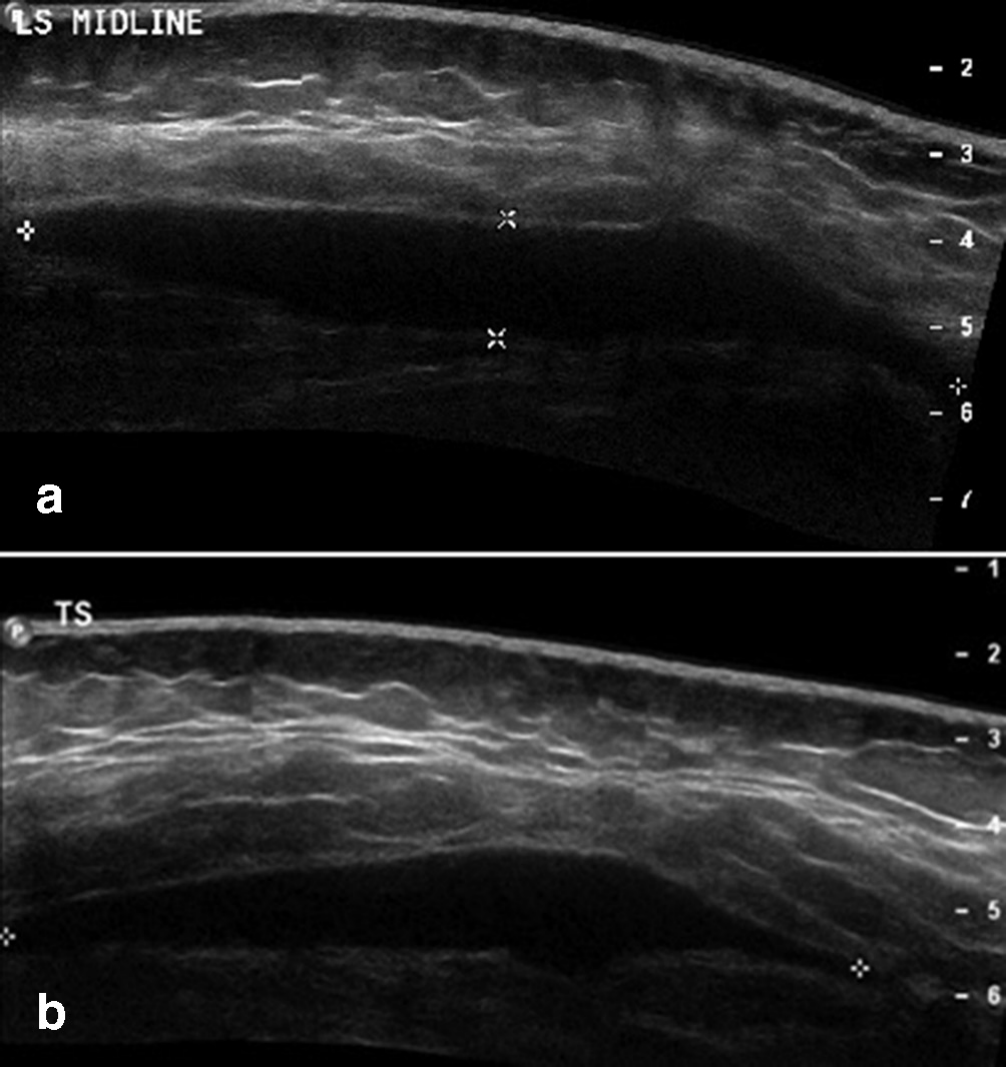
(a) Longitudinal and (b) transverse initial ultrasound of retrosacral Morel-Lavallée lesion at presentation, prior to aspiration. There is a hypoechoic focal fluid collection lying between the deep margin of the subcutaneous fat and the underlying fascia.

Initial ultrasound (Figure 1a and b) demonstrated a hypoechoic, fluctuant and partially compressible fluid collection measuring 10 cm x 10.9 cm x 1.4 cm, lying between the subcutaneous fat and the underlying fascia, superficial to the sacrum. There was no internal debris or solid component. The diagnosis of a Morel-Lavallee type lesion was made and aspiration was recommended.

## Treatment

Three subsequent percutaneous ultrasound-guided aspirations of the Morel-Lavallée lesion were performed over a period of 7 weeks with 110, 30 and 8 ml of serous fluid obtained respectively followed by use of a compression garment. The collection was drained to dryness on each occasion but subsequently recurred with ongoing symptoms.

**Figure 2.**
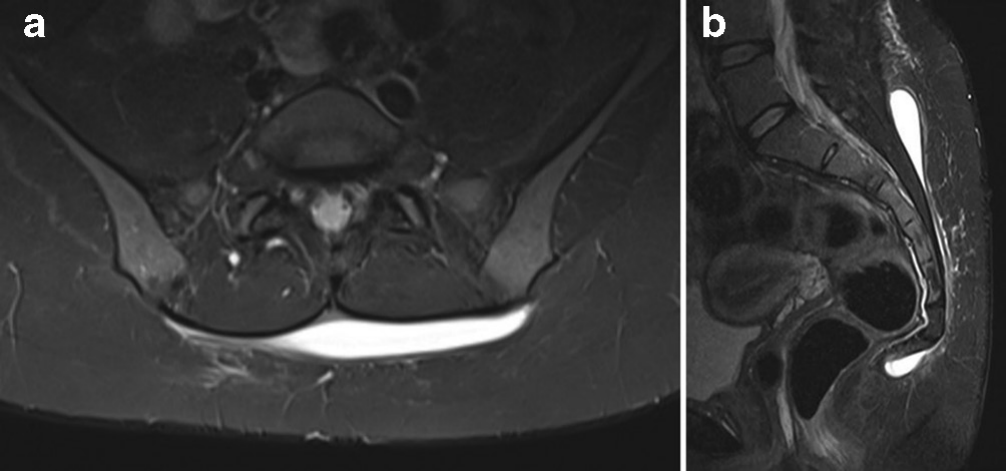
(a) Axial and (b) sagittal initial short tau inversion recovery (MRI) of retrosacral Morel-Lavallée lesion following three percutaneous aspirations. There is a high signal collection seen at the deep margin of the subcutaneous fat, but superficial to the fascia overlying the paraspinal musculature. On the sagittal sequence distal extension of the lesion to the level of the coccyx is identified.

Following the third aspiration, recurrence of a superficial seroma was confirmed with MRI (Figure 2a and b). On this MRI there was a high short tau inversion recovery signal collection seen between the deep margin of the subcutaneous fat and the fascia overlying the parasacral musculature. This extended over the posterior sacrum and distally as far as the coccyx. However, no deep subfascial collection, cerebrospinal fluid communication or underlying occult fracture was demonstrated. A further ultrasound-guided aspiration was performed with an 18 gauge sheathed needle yielding 10 ml of serous fluid, and 5 ml injectable liquid doxycyline (100 mg per 5 ml) was injected into the lesion. The compression garment was then reapplied for 7 days.

## Outcome

**Figure 3.**
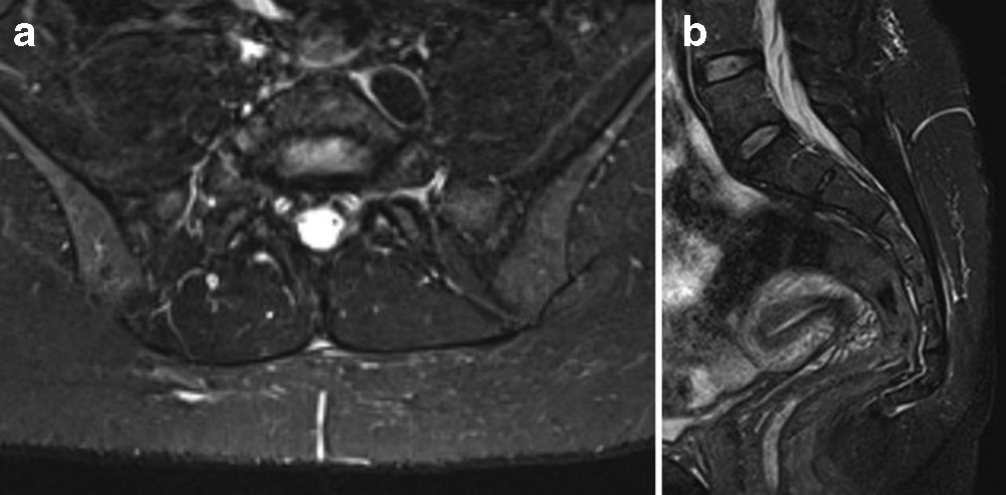
(a) Axial and (b) sagittal short tau inversion recovery (MRI) of sacrum post-sclerotherapy showing no recurrent fluid collection. Trace increased signal at site of previous lesion is likely post-procedural in nature. Sagittal sequence confirms resolution of distal extension of lesion also.

4 months after the final aspiration and instillation of doxycycline, the patient was asymptomatic with resolution of pain and subjective swelling. There were no post-procedure complications. Follow-up MRI (Figure 3a and b) demonstrated only minimal increased signal on a short tau inversion recovery sequence at the previous location of the lesion, with no defined recurrent fluid collection.

## Discussion

Morel-Lavallée lesions are chronic seromas due to closed degloving injuries, resulting from blunt trauma. There is separation of the subcutaneous fat from the underlying fascia with an associated hematoma and subsequent seroma formation.^1^ Granulation tissue at the periphery of the lesion forms a pseudocapsule preventing absorption of the fluid causing a chronic seroma.^2^ Lesions may occur in combination with pelvic and acetabular fractures, but more commonly occur after an isolated soft tissue injury.^3^ Presentation may be delayed by several weeks or months after trauma. Morel-Lavallée lesions commonly occur over the greater trochanteric, gluteal and flank regions,^4^ but have also been reported around the knee and in the lumbar region. A literature review by Vanhegan et al of 204 Morel-Lavallee lesions showed only 3% of these lesions affect the lumbosacral region.^2^ These lesions often fail to regress spontaneously causing ongoing pain and contour deformity. Morel-Lavallée lesions may also be complicated by infection and skin necrosis.

Treatment options include conservative management, surgery, aspiration and sclerotherapy. Acute small lesions are often treated conservatively with compression bandages, non-steroidal anti-inflammatory medication and physiotherapy.^5^ Whilst chronic lesions have traditionally been treated with surgical debridement,^6^ it has subsequently been found that smaller lesions may be amenable to percutaneous aspiration and compression treatment.^7^ Surgery is more invasive and carries risks of viability to the adjacent superficial soft tissue, and it has been suggested that aspiration allows for shorter recovery times.^4^ However, Nickerson et al retrospectively reviewed 87 Morel-Lavallée lesions, 25 of which were treated with aspiration^6^ and they reported a higher rate of recurrence in lesions with over 50 ml aspirated, although it is unclear if repeat aspiration was attempted.

More recently, percutaneous treatment by combined aspiration and sclerotherapy has proved an effective minimally invasive alternative, which has been utilized for hip and knee lesions^8^ as well as in a case study of a lesion extending over the thoracic and lumbar spine.^5^ General complications for aspiration and sclerotherapy are difficult to define given that a heterogenous group of different sclerosants have been trialled, such as talc, ethanol, fibrin glue and tetracycline antibiotics.^9^ With talc sclerodesis, a case series has demonstrated pain during the procedure, as well as a case of infection and subsequent recurrence.^8^ For doxycycline sclerodesis, as with any subcutaneous injection, there are potential risks of pain and infection, with an additional risk of recurrence in Morel-Lavallée lesions, but systematic review of sclerotherapy in seromas has demonstrated no significant complications of doxycycline use.^9^ For use specifically in Morel-Lavallée lesions, Tejwani et al described a series of 27 knee lesions, 13 of which were treated with at least 1 aspiration and 3 repeatedly recurring cases which were successfully treated with doxycline sclerotherapy.^4^ Although several cases recurred after initial aspiration, subsequent sclerosis with doxycycline resulted in no further recurrence. There were no significant complications after doxycycline treatment. Bhansal et al described a series of 16 Morel-Lavallée lesions, which were managed with both aspiration and sclerotherapy. This series reported clinical resolution of lesions following doxycycline injection with compression bandages.^10^ Three of their patients had some short-term post-procedural pain and fever. Changes in skin mobility were described as a frequently occurring complication and one patient reported an inability to run long distances but there was no neuropathy or infection. Neither of these case series demonstrated any serious long-term complication or recurrence relating to doxycycline sclerotherapy. A potential specific complication of doxycycline injection is that outside of treatment for Morel-Lavallée lesions there have been rare cases of neuropathy associated with doxycycline injection used for treatment of pediatric lymphatic malformation of the neck,^11^ with the suggestion that care is taken in use near critical structures. Given that Morel-Lavallée lesions occur between the subcutaneous fat and fascia, rather than adjacent to the vascular and neural structures of the neck, this is less likely to be relevant to sclerotherapy for Morel-Lavallée lesions but should be considered before treatment.

To our knowledge, this is the first reported case of a retrosacral Morel-Lavallée lesion which has been successfully managed with ultrasound-guided aspiration and sclerotherapy with doxycycline. This case demonstrates the value of combined use of MRI and ultrasound in characterizing lesions, and excluding associated complications, as well as image-guided minimally invasive percutaneous therapy for refractory Morel-Lavallée lesion in an atypical location, which had failed to resolve with sequential aspiration alone.

## Learning points

- Morel-Lavallée lesion is a potential cause of persistent post-traumatic superficial soft tissue swelling.
- Morel-Lavallée lesion can be diagnosed with ultrasound but may be better characterized and localized using MRI, especially if in an atypical location.
- Morel-Lavallée lesions can occur in the retrosacral area where traditional management by compression bandage may be less effective. The radiologist may therefore have a role in treating lesions in atypical locations with aspiration and sclerotherapy.
